# Effects of neuromuscular training and strengthening of trunk and lower limbs muscles in women with Patellofemoral Pain: A protocol of randomized controlled clinical trial, blinded

**DOI:** 10.1186/s13063-019-3650-7

**Published:** 2019-10-11

**Authors:** Natália Camin Silva, Matheus de Castro Silva, Morisa Garcia Guimarães, Manoela Beatriz de Oliveira Nascimento, Lilian Ramiro Felicio

**Affiliations:** Programa de Pós-Graduação em Fisioterapia na Universidade Federal de Uberlândia (UFU), Faculdade de Educação Física e Fisioterapia (FAEFI) , Rua Benjamin Constant, 1286, Uberlândia, MG CEP: 38400-678 Brazil

**Keywords:** Patellofemoral pain, Muscle strength, Neuromuscular training, Kinematics, Hip and knee

## Abstract

**Background:**

Patellofemoral pain (PFP) often affects young women, and the etiology is multifactorial and poorly understood. Conservative intervention has been focused on risk factors or aggravating factors and is composed of hip- and knee-strengthening exercises, as this population often has muscle weakness and poor motor control during daily and sports activities. The objective of this study was to evaluate the additional effects of neuromuscular training in a conservative treatment of trunk-, hip-, and knee-muscle strengthening on pain, function, and kinematics of the trunk, pelvis, and lower limb in women with PFP.

**Methods:**

This is a randomised clinical trial, controlled, blinded. Ninety women who are active and engage in physical activity up to twice a week will be recruited. All participants will undergo an individual physiotherapy assessment and then will be allocated randomly into two groups. Thereafter, both groups will undergo a 12-week intervention protocol: group 1 will perform strengthening exercises for the trunk, hip, and knee muscles, while group 2 will receive the same treatment, with the inclusion of neuromuscular training exercises on the fourth week. At the end of the intervention, the volunteers will be evaluated. The primary outcomes will be pain intensity (using a Visual Analog Scale: over the last month, squat 90°, and step of 26 cm during 1 min), functional capacity (Anterior Knee Pain Scale and Activities of Daily Living Scale), and 2D kinematics of the trunk, pelvis, and lower limb during the single-leg squat. The secondary outcomes correspond to the isometric muscular strength of the lower limb and the level of satisfaction from the intervention.

**Discussion:**

The present study was initiated on 28 January 2018 and is currently in progress, scheduled for completion in July 2019. The results of this study should contribute to the physiotherapeutic treatment of women with PFP by aggregating information on the benefits of adding neuromuscular training to strengthening of the trunk and lower-limb muscles.

**Trial registration:**

Registro Brasileiro de Ensaios Clínicos, ID: RBR-8c7267. Registered on 2 August 2017.

**Electronic supplementary material:**

The online version of this article (10.1186/s13063-019-3650-7) contains supplementary material, which is available to authorized users.

## Background

Patellofemoral pain (PFP) is a common musculoskeletal impairment, with high incidence, involving adolescents, military personnel, and elite athletes [1], with an annual prevalence of 22.7% of the general adult population [1], being higher in women [1]. It is defined by pain in the anterior region of the knee, which is accentuated by performing activities that increase the load on the patellofemoral joint, such as squatting and going up and down the stairs [2, 3]. It is believed that PFP is a potentially contributing risk factor for the development of patellofemoral osteoarthritis [4–6].

The etiology of PFP is very complex, multifactorial [7, 8] and seems to involves the biopsychosocial aspects of each individual [9]. It is known that the population with PFP presents anxiety, depression, catastrophism, and fear of movement of the painful joint, and these are correlated with pain [10]. However, more studies are needed to better understand the role of psychological, social, cultural, and behavioral aspects in the development of PFP. In general, the biological factors are associated with abnormal joint load/stress [8].

According to a recent systematic review and meta-analysis of prospective studies [7], isometric/isokinetic weakness of the quadriceps is a strong risk factor for the development of PFP, and moderate evidence indicates that increased strength of the hip abductors is a predictor of PFP in adolescents. However, because of the complexity of PFP, prospective longitudinal studies are still needed to identify possible risk factors [7, 11].

It is known that the population with PFP has a deficit of strength and motor control of the trunk [8, 12–14], posterolateral musculature of the hip [8, 12–17], and quadriceps [7, 8, 12, 18]. Some authors indicate a probable relationship between PFP and lack of appropriate control of lower-limb movement especially in strenuous activities [8, 14, 19–24], in which this motor-control deficit causes excessive dynamic valgus, increasing the lateralization of the patella and, thus, patellofemoral stress [25, 26]. Thus, excessive dynamic valgus is a potentially predictive factor for PFP [26, 27]. In view of this, the use of a protocol that aims to improve the kinematics of the lower limb is indicated, thus minimizing patellofemoral stress [24].

Conservative treatment for PFP has been focused on trunk- and hip-muscle-strengthening protocols associated with strengthening of the knee musculature [28–33], as pain reduction and motor function improvement were observed in these patients [28, 29, 34].

In view of the kinematic characteristics of this population, the integration of neuromuscular training to the strengthening protocol may be important. However, the evidence-base is poor and unclear [32, 33, 35–37]. We found only five studies that evaluated these phases of physical therapy in patients with PFP. One was a case report [32]; two studies observed the effect of motor control under dynamic alignment during gait [35, 36]; one study evaluated trunk and lower-limb kinematics following an isolated knee-strengthening and stretching program versus a hip-strengthening program associated with neuromuscular training [33]. Moreover, only one study evaluated the additional effect of neuromuscular training, concluding that the addition of neuromuscular training does not promote significant improvement in the kinematics of the trunk and lower limb [37]. However, the small sample size, relatively unchallenging motor-control protocol, and short, 4-week, intervention period could have influenced the results [37]. Therefore, ,studies that address neuromuscular training in the treatment of individuals with PFP are necessary.

Taking into account these aspects, the objective of this study was to evaluate the addition of neuromuscular training to the strengthening of the trunk, hip, and knee muscles on pain, functional capacity, and kinematics of the trunk, pelvis, and lower limb in patients with PFP. Our hypothesis is that, compared to the group that received only strength training, the group submitted to the protocol that combined neuromuscular strengthening and training would show greater improvement in pain, function, lower-limb and trunk kinematics, and strength of the hip and knee.

## Methods

### Study design

**Fig. 1:**
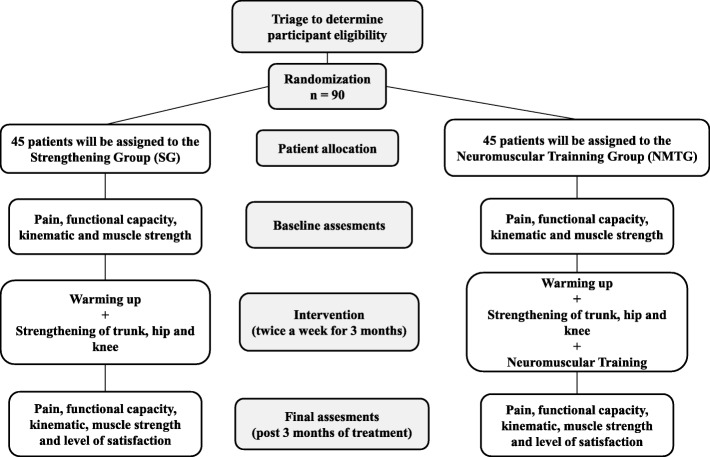
Flowchart of the study design. (Figure adapted according to the model used by Dos Anjos Rabelo ND, et al. [37]. Authorization granted by the authorship and original publisher.)

**Fig. 2:**
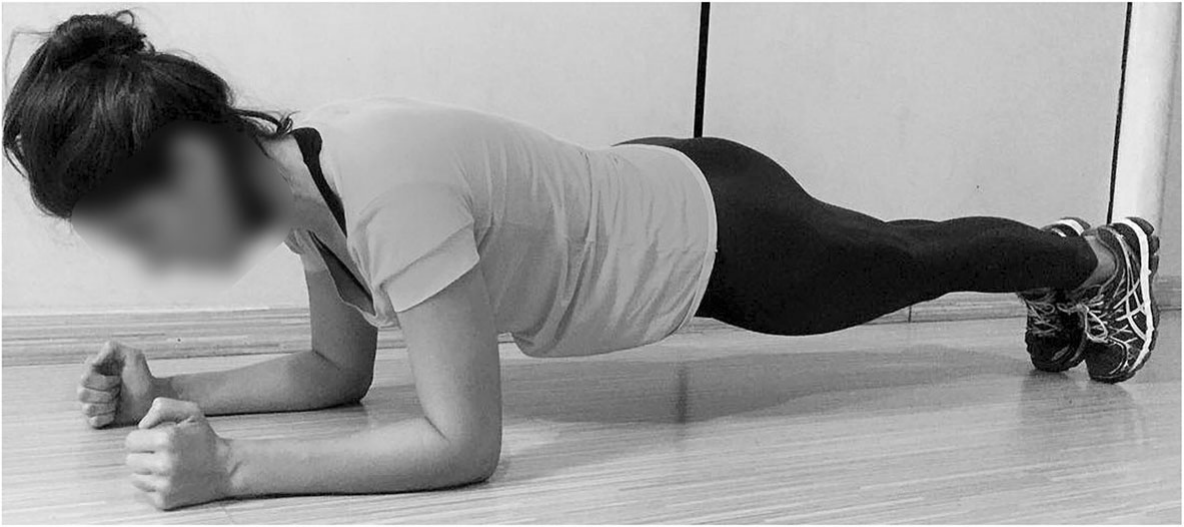
Ventral Plank on a stable surface (1st–12th week). This exercise will be performed by the Strengthening Group (SG) and Neuromuscular Training Group (NMTG). (Originals photos and own authorship)

The present study is a blinded, randomized controlled, clinical trial with two parallel groups (Figs. 1 and 2). The trial was approved by the Human Rights Ethics Committee of the Universidade Federal de Uberlândia (UFU) under protocol number CAAE: 57621316.0.0000.5152. The study was registered at Registro Brasileiro de Ensaios Clínicos (ReBEC) (trial registration number, RBR-8c7267) and is being funded by the Coordenação de Aperfeiçoamento de Pessoal de Nível Superior (CAPES). It started recruiting patients on 28 January 2018, and data collection will likely be completed by July 2019. The evaluations will be performed before and after the 12-week intervention, and the variables observed are as follows: pain intensity, functional capacity, kinematics of the trunk, pelvis, lower limbs, and muscle strength.

### Participants and therapists

The sample size calculation was performed using the mean difference and standard deviation error (based on outcomes of pain; function; kinematics of the trunk, hip, and lower limb during the single-leg squat; and strength of the hip and knee) of similar studies in the literature. The power of the test considered was 80% and the alpha was 0.05, with a sample size of 45 per group. In this analysis, the program BioEstat version 5.3 (Manaus, Brazil) was used.

A total of 90 active female individuals aged 18–30 years who have anterior or retropatellar pain in the knee (with a Visual Analog Scale (VAS) score ≥ 3) [2, 33] for at least the last 3 months (in two or more of the following activities: running, walking, jumping, climbing up and/or down stairs, remaining seated or kneeling for a long period of time, squatting, during isometric extension of the knee with 60° of flexion, and during palpation of the medial or lateral facet of the patella) [33], and who perform any type of physical activity up to twice a week and/or are irregularly active A and B individuals according to the International Physical Activity Questionnaire (IPAQ), will be recruited [38]. The exclusion criteria are as follows: previous physical therapy treatment; history of knee surgery; patellar dislocation; ankle or hip injuries; meniscal or ligament injury; any other specific knee changes; tendinitis in the lower extremity; low back pain; sacroiliac joint pain; pregnancy; and the presence of any neurological, cardiovascular, and rheumatological changes or any changes that compromise the understanding of the procedure and affect physical therapy tests and treatment [15, 31].

These individuals will be recruited through posters set up on the campus of the Universidade Federal de Uberlândia (UFU), and by dissemination via social networks and radio in the city of Uberlândia, with the purpose of informing and arousing the participatory interest in the desired population.

This research will be developed in the Laboratório de Avaliação em Biomecânica e Neurociências (LABiN) and the Physical Therapy Clinic School of UFU, Brazil.

### Procedure

All subjects will receive a written Consent Form to participate in this study. Thereafter, the participants will be submitted to an individual physiotherapeutic evaluation, and if they meet the inclusion criteria they will be randomly distributed into two intervention groups.

The randomization (fixed allocation of the simple type) will be conducted by an investigator who is not involved in the recruitment, evaluation, and/or treatment of participants. The randomization codes will originate from the RAND function of Microsoft Excel for Windows and will be inserted into sealed opaque envelopes and listed sequentially to hide the allocation. The envelopes will be opened by the same investigator who generated the codes.

The investigator responsible for the physiotherapeutic evaluation is blinded to the treatment allocation. Participants will be informed that they will receive physiotherapeutic treatment but will not know of the differences between the treatment groups, or the study hypothesis, and can be considered as blinded. Three physiotherapists were adequately trained to apply the intervention protocol. Each patient will be treated by only one therapist who is not involved in the physiotherapeutic evaluation and can be considered as blinded.

### Intervention/control

Ninety patients will be randomly allocated into two groups: (1) Strengthening Group (SG): submitted to strengthening exercises of the muscle trunk, hip, and knee; and (2) Neuromuscular Training Group (NMTG): submitted to the same protocol as the SG, with the addition of neuromuscular exercises on stable and unstable ground.

Both groups will be submitted to physiotherapeutic treatment for a period of 12 weeks, performing two sessions per week, totaling 24 sessions, with a duration of 60–90 min per session. Each session will consist of 10 min of warming up on elliptical equipment, with a comfortable velocity for the patient and mild-moderate intensity, followed by protocol exercises, as described (see Additional file 1).

Mandatorily, during the first 2 weeks of treatment and/or in the presence of pain, the proposed exercises will be performed within the “protection range” of the patellofemoral joint, with 90–45° of knee flexion during exercises in Open Kinetic Chain (OKC) and 0–45° of knee flexion during Closed Kinetic Chain (CKC) exercises [39]. With the exception of squatting (“wall squat”), it will be performed between 0° and 60° of knee flexion [40]. From the third week, the range of motion of knee flexion will be progressed (in exercises “seated knee extension,” “leg press,” and “wall squat”). This progression will only be performed when the patient has no pain (VAS score 0) or feels only discomfort (VAS score 1) above the protection range of the patellofemoral joint. If the patient experiences pain (VAS score 2 or more) when progressing through the range of motion, the patient will be asked to perform the exercise as much as possible in a pain-free or discomforting (VAS score 0–1) range of motion. If unable to increase the range of motion the exercise will be kept within the protection range of the patellofemoral joint (90–45° for OKC; and 0–45° for CKC) until the pain has resolved.

The initial load during training will be standardized at 70% of 1 repetition maximum (1RM) – defined as the maximum load at which a patient can perform only one repetition of the exercise with good quality and without pain or the need to compensate. One repetition maximum of the exercises will be determined on the first day of care and revised each week for possible adjustments and changes in the load for all exercises. Only the “wall squat” exercise will be started with a load corresponding to 10% of the body weight of each patient.

Exercise loads will be increased weekly starting from the third week. There is a standard progression model, which corresponds to a 5–10% increase in current load. The criteria used for progression are: (1) the presence of full range of motion with VAS score 0–1; (2) performing the same exercise without the need to compensate. These criteria were established based on the protocol described by Baldon et al. [33]. CarciBAND® elastic bands (São Paulo, Brazil), a balance disc from ACTE Sports® (www.actesports.com), model T6-AZ, and a Physicus® (Neighborhood Limoeiro) trampoline will be used in this study.

The exercise program for both groups will consist of three sets of 10 repetitions. The exercise on the “board” will be performed with three repetitions in which the patient should maintain the posture for a maximum time that can be achieved. After each set, a rest interval of 1–2 min will be established. In the presence of moderate to severe pain during exercise (minimum VAS score 5), the session will be interrupted and analgesic measures will be performed immediately.

All patients will be instructed not to perform any other type of treatment for knee pain and to maintain their daily life activities without performing any resisted lower-limb exercise outside the study sessions.

All patients will be informed of inappropriate movement changes in the lower limb during the exercises. In addition, they will be educated to correct any alignment of the lower limb during the exercises through verbal commands and visual feedback.

The minimum rest period between the weekly sessions will be about 48 h.

### Outcome measures

In this study, four outcome measures evaluated before and after the intervention will be used.

### Primary outcomes

The primary outcomes correspond to the pain intensity measured by the VAS in three domains: over the last month, squat 90°, and step of 26 cm during 1 min); functional capacity through two questionnaires: the Anterior Knee Pain Scale (AKPS) and the Activities of Daily Living Scale (ADLS); and two-dimensional (2D) kinematics of the trunk, hip, and lower limb during the single-leg squat.

### Secondary outcomes

The secondary outcomes correspond to the maximum isometric muscle force measured by the manual dynamometer (Lafayette Instrument Company, Lafayette, IN, USA) and the level of satisfaction of the patient in relation to the treatment received, through two multiple choice questions.

Each outcome measure is described below:

- Pain assessment

Pain will be assessed through the VAS [41, 42], with a scale of 0 (without pain) to 10 (extreme pain). The VAS will be applied considering two moments: (1) pain in the last month (current moment); and (2) before and after performing two functional activities: bilateral squatting at 90° (for 1 min) and step-up/down 26 cm (for 1 min).

The VAS is responsive, sensitive and valid to evaluate the PFP population [41, 42].

- Functional assessment

To evaluate function, we will use the ADLS [43–45] and the AKPS [44, 46, 47] questionnaires. These instruments are considered reliable, responsive and valid for the population with PFP [44, 47]. The ADLS consists of three questions that measure the individual’s overall function level on a scale of 0–100. In addition, it has 14 items, which generally measure the symptoms and functional limitations caused by PFP in daily life activities. Moreover, AKPS has 13 items, which also evaluate the symptoms and functional limitations that are often present in individuals with PFP. In both questionnaires, the score ranges from 0 to 100, where 0 corresponds to the greatest functional impairment in relation to pain and 100 indicates no functional impairment.

- Kinematic assessment

In the frontal and sagittal planes, the lower-limb kinematics will be evaluated during squatting [14, 22, 24, 33] through 2D shooting with two full HD camcorders (JVC GZ-E10, JVC, Wayne, NJ, USA). The cameras will be positioned frontal and lateral to the participant, at a distance of 2.5 m [48]. Evaluation of both lower limbs will be performed.

The static calibration of the system will be performed using a 160-cm-long stick. Subsequently, the patient’s bipodal static position will be recorded.

Self-adhesive labels will be positioned bilaterally in the following points: lateral malleolus and medial and anterior tibial tuberosities, lateral and medial epicondyles of the femur and anterior superior iliac spine, upper region of the iliac crest, and spinous process of the vertebra (chest T7, cervical vertebra C7, and the centre of the sternum).

To perform the single-leg squat test, the participants will remain in unipodal support with the contralateral limb to the side with the knee flexed at 90° and arms positioned at the waist. Deep squats will be performed without the contralateral foot touching the ground. The minimum squat angulation is 60°. If, during the test, some of the requirements are not fulfilled, the test will be invalidated and repeated.

For familiarization, the participant will be asked to squat twice with each leg, with a rest interval of 2 min between each movement. After familiarization, the participants will perform the single-leg squat three times consecutively, in which the average of the measured angles is considered for analysis. In the frontal plane, the following angles will be measured: dynamic valgus of the knee, pelvic tilt, and lateral flexion of the trunk. In the axial planes, the angles of anterior trunk flexion and knee flexion will be measured. The analysis of these angles will be performed by the Kinovea® program.

- Strength evaluation

The measurement of the strength of knee extensors [49], abductors [15], lateral rotators [15], and hip extensors [15] will be obtained by means of an evaluation using the manual dynamometer (Lafayette Instrument Company, Lafayette, IN, USA) [50, 51]. An instrument that is valid and reliable [50].

To measure the strength of the knee extensors, the individual will be seated at the edge of the stretcher with the hips flexed at 90° and the knee to be tested at 60°. For the abductors, the individual will be positioned lying on their side, and the limb to be tested will be facing upwards, with neutral rotation of the hip, 10° of extension, and 20° of abduction [15]. For the lateral rotators, the individual will be seated at the edge of the stretcher with hips and knees flexed at 90° and will be oriented to perform slight lateral rotation so that the lateral malleolus is aligned with the midline of the body [15]. For the hip extensors, the individual will be in the prone position with the knee to be tested having 90° of flexion and 10° of extension [15].

The use of a stabilizing strap will be adopted during the tests to avoid the need to compensate, and to stabilize the dynamometer. Before performing the tests, the patient will be asked to perform two submaximal isometric contractions of each muscle group, for familiarization, with a 1-min rest to start the test. The patient will be asked to perform three maximum voluntary isometric contractions of each muscle group for 5 s. A 1-min rest period will be allowed between each measurement. During the conduct of all tests, the patient will be verbally encouraged using the words “Force, force, force!” For the analysis, we will consider the average strength of the test of each muscle group.

- Level of satisfaction

This parameter will be measured during the reassessment of the patient, using a qualitative questionnaire composed of two multiple choice questions, elaborated exclusively by the principal investigator of the study. The purpose of this questionnaire was to measure the patient’s satisfaction with the treatment and their current clinical condition [6].

### Data analysis

The Kolmogorov-Smirnov normality test will be performed. If the null hypothesis is confirmed, parametric data for the comparisons will be used. However, if the null hypothesis is not confirmed, nonparametric tests will be conducted.

For parametric data, comparison between the groups will be carried out using linear mixed models, considering *P* values ≤ 0.05 as significant difference. For intragroup comparison (pre and post intervention), the analysis of variance (ANOVA) test will be used for repeated measurements, considering *P* values ≤ 0.05 as significant difference.

The clinical relevance of the results will be confirmed by calculating the effect size (Cohen’s d) of the significant differences. The following effects will be considered: 0.00–0.49, small; 0.50–0.79, medium; and above 0.80, large (Cohen, 1988). An intention-to-treat analysis will be performed [52].

## Discussion

Although the protocol for strengthening the hip and knee muscles has already been well established in the literature and is considered “gold standard” conservative treatment in individuals with PFP [34] because of its effectiveness in improving pain, function, and the kinematics of the lower limb [34], the effects of neuromuscular training on the pattern of lower-limb movement and the addition of these exercises in the physiotherapeutic treatment program remains less well discussed and understood [37].

In view of this, the results of the present study may contribute to the decision-making for the physiotherapeutic intervention of patients with PFP by providing information on the effects of neuromuscular training on the clinical and kinematic conditions in this population Additional file 2.

## Additional files

Additional file 1: This additional file describes in detail the exercises of the proposed protocol for both study groups. (DOCX 21 kb)

Additional file 2: Standard Protocol Items: Recommendations for Interventional Trials (SPIRIT) 2013 Checklist: recommended items to address in a clinical trial protocol and related documents*. (PDF 119 kb)

## Data Availability

Data sets generated and/or analyzed during the current study may be made available by the corresponding author upon reasonable request.

## Abbreviations

ADLS: Activities of Daily Living Scale
AKPS: Anterior Knee Pain Scale
CAPES: Coordenação de Aperfeiçoamento de Pessoal de Nível Superior
NMTG: Neuromuscular Training Group
PFP: Patellofemoral pain
SG: Strengthening Group
UFU: Universidade Federal de Uberlândia
VAS: Visual Analog Scale
