# Supplementary material for: Effects of neuromuscular training and strengthening of trunk and lower limbs muscles in women with Patellofemoral Pain: A protocol of randomized controlled clinical trial, blinded
Source: Trials. 2019 Oct 11;20:586. doi: 10.1186/s13063-019-3650-7 (PMC6788005; doi:10.1186/s13063-019-3650-7)
Supplement: Supplementary file 1 — This additional file describes in detail the exercises of the proposed protocol for both study groups. (DOCX 21 kb) [file 13063_2019_3650_MOESM1_ESM.docx]

**Additional File 1. Protocol exercise table (.docx)**

| Exercises | Description/Progression |
| --- | --- |
| Warming up | Elliptical, no load, with comfortable velocity to patient and mild-moderate intensity for 10 min. |
| 1. Ventral plank on a stable surface  (1st–12th week) | In ventral decubitus position, shoulders and elbows are flexed at 90º (forearm support in the ground). This position should be kept for as long as possible.  **Sets:** 3  **Progression:** Increased posture maintenance time |
| 2. Wall squat  (1st–12th week) | Standing, lower limbs parallel to shoulder width. Instructed to squat up to 60º of knee flexion.  **Sets/Repetitions:** 3×10  **Load/Resistance:** The initial load will correspond to 10% of the patient’s body weight. The load will be in the anterior region near the chest.  **Progression:** Increase of 5–10% of the load weekly |
| 3. Lateral walk  with elastic band  (1st–12th week) | Standing, lower limbs parallel to shoulder width, knee flexed at 60°. The patient will be guided to walk to the side.  **Sets/Repetitions:** 3×6 for each side  **Load/Resistance:** Elastic band around the distal region of the thigh  **Progression:** Increase of 1 gauge of elastic band, according to:  - 1st and 2nd week: strong average  - 3rd–5th week: strong  - 6th–8th week: super strong  - From the 9th week: extra strong |
| 4. Hip extension in  Prone position  (1st–12th week) | In the ventral decubitus position, the patient will be oriented to extend the hip keeping the limb neutral and the knee extended. If the patient cannot perform the exercise in this way, it will be initially performed with the knee flexed at 90º.  **Sets/Repetitions:** 3×10  **Load/Resistance:** Ankle weights in the distal region of the leg. Initial loading of 70% of 1RM.  **Progression:** Increase of 5–10% of the load weekly, if able. |
| 5. Leg Press –  0 –45º  (1st–12th week) | Sitting, lower limbs parallel to shoulder width. The patient will be oriented to bend the knee up to 45º of flexion.  **Sets/Repetitions:** 3×10  **Load/Resistance:** Initial load of 70% of 1RM  **Progression:** Increase of 5–10% of the load weekly, if able. |
| 6. Seated knee  extension  (1st–12th week) | Sitting, hips and knees bent at 90°. The patient will be instructed to extend the knees up to 45º of knee flexion.  **Sets/Repetitions:** 3×10  **Load/Resistance:** Initial load of 70% of 1RM  **Progression:** Increase of 5–10% of the load weekly, if able. |
| 7. “Clam”  (1st–12th week) | In side-lying position, hips and knees are flexed at 60º and 90º, respectively. The patient will be instructed to abduct and rotate the femur laterally.  **Sets/Repetitions:** 3×10  **Load/Resistance:** Elastic band around the distal region of the thigh.  **Progression:** Increase of 1 gauge of elastic band, according to:  - 1st week: strong average  - 2nd–3rd week: strong  - 4th and 5th week: super strong  - From the 6th week: extra strong |
| 8. Pelvic drop in  standing  (≅ 6th–12th week) | Member supported on one step and the other suspended, pelvis and neutral trunk. The patient will be instructed to slowly lower the pelvis (from the suspended limb) toward the floor, keeping the knees extended and feet neutral and return to the starting position without compensation.  **Sets/Repetitions:** 3×10  **Load/Resistance:** Ankle weights in the distal region of the leg. Initial loading of 70% of 1RM.  **Progression:** Increase of 5–10% of the load weekly, if able.  **Note:** This exercise will be progression of the “Clam” exercise. Moreover, it will only start after the 6th week, if the patient has enough strength and is able to perform it correctly. When the patient can do so, exercise 8 will replace exercise 7 |
| Treatment protocol exclusive to the Neuromuscular Training Group (NMTG) | |
| 9. Ventral plank  on an unstable  surface  (4th–12th week) | In ventral decubitus position, shoulders and elbows are flexed at 90º under the balance disc. This position should be kept for as long as possible.  **Sets:** 3  **Progression:** Increased posture maintenance time. In addition, modification of the unstable surface according to:  - 4th–6th week: both elbows on 1 balance disc (Figure 10A)  - 7th–9th week: each elbow on a balance disc, separately (Figure 10B)  - 10th–12th week: both elbows on the same balance disc and both feet under balance disc (Figure 10C)  **Note:** This exercise will replace ventral plank exercise on a stable surface. |
| 10. Lunge with  elastic band  (4th–12th week) | Standing, one leg in front of the body. The patient will be oriented to crouch so that the anterior knee reaches 60º of flexion and the knee of the posterior leg reaches approximately 75º of flexion and return to the home position.  **Sets/Repetitions:** 3×10  **Load/Resistance:** Elastic band around the knee causing force toward the valgus, inducing abduction and lateral rotation of the hip.  **Progression:** Increase of 1 gauge of elastic band, according to:  - 4th–6th week: strong average  - 7th–9th week: strong  - 10th–12th week: super strong |
| 11. Single-leg  squat on a stable  surface  (4th–7th week) | Standing on the ground, the patient will be instructed to crouch unipodal until 60º of knee flexion, keeping the contralateral limb in front, with flexed hip and extended knee.  **Sets/Repetitions:** 3×10  **Load/Resistance:** Elastic band around the knee causing force toward the valgus, inducing abduction and lateral rotation of the hip.  **Progression:** Increase of 1 gauge of elastic band, according to:  - 4th and 5th week: without elastic band (Figure 12A)  - 6th week: medium strong band (Figure 12B)  - 7th week: strong band (Figure 12B) |
| 12. Single-leg  squat on unstable  surface  (8th–10th week) | Standing on the trampoline, the patient will perform the exercise described above (number 11).  **Sets/Repetitions:** 3×10  **Note:** This exercise will replace the single-leg squat on a stable surface. |
| 13. Single-leg squat on an unstable surface with elastic band  (11th–12th week) | Similar to exercise 12, adding stress on valgus provided by an elastic band. Patient will be instructed to maintain the knee without medial deviation.  **Sets/Repetitions:** 3×10  **Load/Resistance:** Elastic band around the knee causing force toward the valgus, inducing abduction and lateral rotation of the hip.  **Progression:** Increase of 1 gauge of elastic band, according to:  - 11th week: strong  - 12th week: super strong  **Note:** This exercise will replace the single-leg squat on an unstable surface |
| General Note | The neuromuscular exercises (9 to 13) will be performed only by the NMTG and will only be added to the session at the beginning of the 4th week. Exercises 12 and 13 will be progressions for exercise 11 and will only begin when the patient is able. |
